# Hazard Ranking Method for Populations Exposed to Arsenic in Private Water Supplies: Relation to Bedrock Geology

**DOI:** 10.3390/ijerph14121490

**Published:** 2017-12-01

**Authors:** Helen Crabbe, Tony Fletcher, Rebecca Close, Michael J. Watts, E. Louise Ander, Pauline L. Smedley, Neville Q. Verlander, Martin Gregory, Daniel R. S. Middleton, David A. Polya, Mike Studden, Giovanni S. Leonardi

**Affiliations:** 1 Environmental Epidemiology Group, Centre for Radiation, Chemical and Environmental Hazards, Public Health England (PHE), Chilton, Oxfordshire OX11 0RQ, UK; tony.fletcher@phe.gov.uk (T.F.); Rebecca.close@phe.gov.uk (R.C.); middletond@fellows.iarc.fr (D.R.S.M.); michael.studden@gmail.com (M.S.); giovanni.leonardi@phe.gov.uk (G.S.L.); 2 London School of Hygiene and Tropical Medicine, Keppel Street, London WC1E 7HT, UK; 3 Inorganic Geochemistry, Centre for Environmental Geochemistry, British Geological Survey, Nottingham NG12 5GG, UK; mwatts@bgs.ac.uk (M.J.W.); land@bgs.ac.uk; (E.L.A.); pls@bgs.ac.uk (P.L.S.); 4 Statistics, Modelling and Economics Department, PHE, 61 Colindale Avenue, London NW9 5EQ, UK; neville.verlander@phe.gov.uk; 5 Cornwall Council, Environmental Protection Team, Public Health and Protection, Camborne, Cornwall TR14 8SX, UK; mgregory@cornwall.gov.uk; 6 School of Earth and Environmental Sciences, and Williamson Research Centre for Molecular Environmental Science, University of Manchester, Manchester M13 9PL, UK; david.polya@manchester.ac.uk

**Keywords:** arsenic, private water supplies, geology, public health risk, hazard and exposure assessment, environmental public health tracking

## Abstract

Approximately one million people in the UK are served by private water supplies (PWS) where main municipal water supply system connection is not practical or where PWS is the preferred option. Chronic exposure to contaminants in PWS may have adverse effects on health. South West England is an area with elevated arsenic concentrations in groundwater and over 9000 domestic dwellings here are supplied by PWS. There remains uncertainty as to the extent of the population exposed to arsenic (As), and the factors predicting such exposure. We describe a hazard assessment model based on simplified geology with the potential to predict exposure to As in PWS. Households with a recorded PWS in Cornwall were recruited to take part in a water sampling programme from 2011 to 2013. Bedrock geologies were aggregated and classified into nine Simplified Bedrock Geological Categories (SBGC), plus a cross-cutting “mineralized” area. PWS were sampled by random selection within SBGCs and some 508 households volunteered for the study. Transformations of the data were explored to estimate the distribution of As concentrations for PWS by SBGC. Using the distribution per SBGC, we predict the proportion of dwellings that would be affected by high concentrations and rank the geologies according to hazard. Within most SBGCs, As concentrations were found to have log-normal distributions. Across these areas, the proportion of dwellings predicted to have drinking water over the prescribed concentration value (PCV) for As ranged from 0% to 20%. From these results, a pilot predictive model was developed calculating the proportion of PWS above the PCV for As and hazard ranking supports local decision making and prioritization. With further development and testing, this can help local authorities predict the number of dwellings that might fail the PCV for As, based on bedrock geology. The model presented here for Cornwall could be applied in areas with similar geologies. Application of the method requires independent validation and further groundwater-derived PWS sampling on other geological formations.

## 1. Introduction

Domestic Private Water Supplies (PWS) provide water used for drinking and cooking to a proportion of the population varying according to the availability of alternative sources and distribution networks and householder attitudes. This proportion is estimated at approximately 1% in the UK [1]; from 10% to 30% in the United States [2], and over 90% in Bangladesh [3,4]. Groundwater (GW) is a common source for PWS, and its contamination by chemicals, of either geogenic or anthropogenic origin, can lead to preventable population exposure to health hazards [5,6,7,8]. In particular, inorganic arsenic is a recognised carcinogen and toxicant [9,10,11] commonly present in GW [12] and arguably the waterborne inorganic chemical with the highest global detrimental impact on public health [13].

Environmental Public Health Tracking (EPHT) can be defined as “the ongoing collection, integration, analysis and interpretation of data about environmental hazards, exposure to environmental hazards, and human health effects potentially related to exposure to environmental hazards. It includes dissemination of information learned from these data and implementation of strategies and actions to improve and protect public health” [14]. A key distinction between EPHT and traditional surveillance is the emphasis on data integration across hazard, exposure and health information systems, therefore an EPHT approach to arsenic (As) in PWS could have an initial goal to characterise and monitor population risk represented by As. This approach has been applied to public health monitoring of As in PWS in the USA [15,16,17,18,19]. In Louisiana, US, the EPHT approach has been used to prioritise parishes for further risk assessment and testing of As in PWS [16]. The EPHT approach could be applied to PWS in other geographical areas [20].

An EPHT programme for prevention of health effects deriving from exposure to As from PWS should consider information that can be obtained from the examination of a possible correlation between bedrock type and As concentration in GW-sourced PWS, as this could contribute to the estimation of population hazard, and related exposure and risk attributable to As. Geostatistical approaches to predicting GW arsenic concentrations have recently been widely implemented [21,22,23,24,25,26] and have been found to be useful particularly in shallow sediment/sedimentary-rock hosted aquifers. Ayotte et al. [27] suggested a possible geological source as a predictor of high As in New England PWS. Bedrock aquifers were reported to have higher As concentrations than unconsolidated aquifers. Arsenic concentrations in water from bedrock wells in variably calcareous metasedimentary rocks were significantly higher (*p* = 0.001) than water in bedrock wells in the combined differentiated metasedimentary and felsic igneous groups [27].

Whilst larger commercial and multi-use PWS in England are subject to risk assessment and testing for compliance to PWS Regulations (2016) [28] by local authorities, by 2016 68% of PWS had been risk assessed [29]. Single domestic PWS are not subject to risk assessment, unless requested by the owner or occupier. Water testing and treatment varies by area and in some regions potential As risk is unknown [29]. 

GW in areas of metalliferous mineralization is known to be particularly vulnerable to release of As. Metal sulphide minerals and their weathering products can release As and other trace elements as a result of mineral oxidation and desorption reactions. Release under anoxic GW conditions as a result of dissolution or desorption from metal oxides has also been well-documented [12]. Cornwall, in south west England, is known to have geologically enhanced As concentrations due to the presence of natural Cu-Sn-Zn-As mineralisation of rocks, which has been locally exacerbated by historical mining and ore processing activities. A water sampling programme of 497 small domestic PWS supplying 508 households was conducted in Cornwall during 2011–2013 and found that up to 35% of supplies had exceedances of one or more prescribed concentration or value (PCV) of a range of chemicals and that 20% of households had one or more exceedance of health-based values for drinking water (DW), including 12% above the manganese PCV [30,31]. Six percent of households failed the As PCV: the PCV and WHO guideline value [32] for As in PWS is 10 μg/L [32,33]. Biomonitoring of residents with PWS confirmed exposure to As through this exposure route, with high concentrations of As found in the urine, hair and toenails of some residents [34,35]. For PWS where DW was greater than 10 μg/L As, a strong correlation between DW As and urinary inorganic As was reported [34].

The confirmation of exposure to PWS As means that characterising As GW hazard, and hence population exposure in England, is an appropriate first step for a national programme of PWS tracking of chemicals in DW. This could subsequently address the related exposure hazard and health risk [36]. 

Hattis and Burmaster [37] argue the case for using distribution-based analysis in exposure assessments to examine variability and uncertainty. A distribution-based approach could be used to explore known relationships between geology and As concentrations in water, and then predict exposure in areas not systematically monitored.

The aim of this paper is to develop a methodology for a population-based hazard assessment and ranking of hazard for As in PWS used for drinking, based on geological bedrock classification. We estimate likely numbers of households at risk and how to best find those at risk based on bedrock geology.

## 2. Materials and Methods

### 2.1. Study Design

We conducted an observational, cross-sectional study of the population of Cornish PWS users. A model of exposure assessment predicts potential dwelling exposure through PWS based on the As distributions measured per simplified bedrock geological classification (SBGC). The method follows the model used to predict radon concentrations in dwellings in the UK by the National Radiological Protection Board (NRPB) [38], subsequently Public Health England (PHE) [39].

### 2.2. Study Population

The population was defined as users of DW originating from single domestic PWS in the county of Cornwall, in south west England, UK. Private well records used for access to users of PWS for domestic use were obtained by PHE in the form of a database compiled by the local authority, according to the provision of the PWS Regulations [28,33]. Residents of Cornwall were eligible; domestic use of the water was the primary focus, although in some instances it was used for other purposes (e.g., farms, dairy, food production, caravan parks or small rural businesses).

### 2.3. Rationale for Simplified Geological Classification

The rationale for the sampling design aimed to lead to an effective public health management approach, by integration of environmental (bedrock) and public health (concerning population groups) information for assessment of water results, as part of an EPHT proof of concept study. A SBGC was devised to approximate the geological features across areas sufficiently large to characterise potential population exposure to chemicals via DW, as described in Ander et al. [30]. The Cornwall area was divided into nine broad geological categories, defined on the basis of rock types (igneous/metamorphic/sedimentary), mineralogical characteristics (e.g., sulphide-bearing/slaty) and stratigraphy (age and therefore likely provenance). In addition to the nine categories, a further specific category was defined as a “mineralised zone” which is represented by the area 1 km around a mapped surface observation/record of Cornubian metalliferous mineralisation from the 1:50,000 scale British Geological Survey (BGS) digital map (available as a “polyline” Geographical Information System (GIS) file). The 1 km buffer zone provided the GIS “polygon” datafile, which was merged with the mapped solid geological information to provide the ten categorised geological groups. The SBGC was attributed to the centroid of each valid postcode in the datafile provided according to the geological classification.

The simplified classification was used for pre-sampling aggregation of the land area because there is a complex sequence of indurated and poorly permeable rocks. The sedimentary rock formations are variable but mainly argillaceous (fine-grained, clay-rich) types. Igneous rocks have compositions ranging from ultramafic to granitic, and may occupy large continuous areas, or be found as thinner layers intruded within (meta) sedimentary rocks. Mineralisation cross-cuts multiple sedimentary and/or igneous geological boundaries. Physical components can also effect GW flow and chemical composition. Weathering and rock structure (such as faulting and fracturing) can create preferential flow paths, facilitating mobilisation and transport of dissolved mass (even in rock units with otherwise very low bulk hydraulic conductivity values). Mineralisation is often associated with these structural features (upward movement of mineralising fluids are frequently associated with deep-seated fault structures). Structural features may create preferential routes and allow contact between moving water and mineralised ground [40].

**Figure 1:**
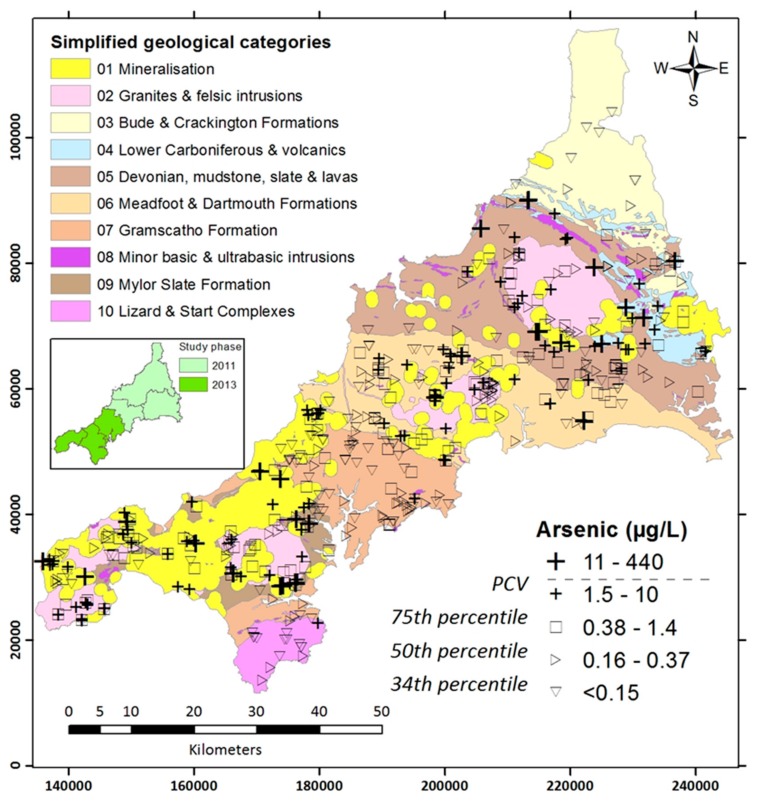
Map of Cornwall showing Simplified Bedrock Geological Classifications (SBGC), location and levels of arsenic measured in Private Water Supplies (PWS) tested. Geological data reproduced with the permission of the British Geological Survey © Natural Environment Research Council (NERC).

These observations meant that a drinking-water sampling strategy for the area should include not only areas of known mineralisation, but also the geological formations represented. Figure 1 shows a map of the sampling area, divided into ten categories on the basis of geological types (basic intrusions are of limited spatial extent and were excluded).

### 2.4. Recruitment of Households Using PWS

The recruitment strategy aimed to reconstruct a population exposure profile for chemicals in water actually consumed by residents. The existing list of households known to use PWS was provided in late 2010 by Cornwall Council and used for two field campaigns, east Cornwall (2011) and west Cornwall (2013). Households were targeted where it was known that they had a GW source (borehole, spring capture or traditional large-diameter well) serving a PWS. Properties using public supplies, PWS surface water sources, or not using the PWS for domestic DW were excluded. A stratified, random design was used: records were classified into one of the SBGC, within which the records were randomised for sequence of contacting. Householders were encouraged to participate by the provision of the measured chemical data for their property, free-of-charge. Contact was made by letter and follow-up phone calls to ensure adequate recruitment. In east Cornwall, a local newspaper advertisement was also used by the county council. After recruitment for each phase, households that volunteered for the study had their PWS DW sampled in spring (March/April) in 2011 or 2013 and measured for As and a broad suite of 55 elements and other chemical parameters, including anions by ion chromatography, pH/alkalinity and conductivity [30].

From the local authority database, a total of 3095 records were retained that met the inclusion criteria. Thirty-three (1%) records were removed due to the use of surface water for the PWS rather than GW. Some records were incomplete, and these were clarified on contact with potential volunteer householders to ensure that the PWS met the study design criteria.

Contact details of residents were incomplete for 37% of the records. Where a valid address could not be established, the record could not be used (~30% of records). Over 2000 letters were sent inviting households to participate. Users of PWS without an appointment who approached the sampling teams in the field were also included, resulting in some convenience sampling where householders were available for water sampling and interviewing (~2%).

On recruitment, a household representative and/or PWS owner were asked a series of questions by telephone, about the nature of the water supply, water treatment methods, the number of people living in the property and the number of properties the supply served. Further details of the recruitment process are given in [30,31,41]. Further chemical data collection methods were detailed in Ander et al. [30]. Where possible, we collected paired GW and DW samples to show the effect of treatment, but this was possible for only 33% of the households. Using only GW results in the modelling would limit the stratification by SBGC, so we modelled distributions based on DW concentrations.

Where more than one property was using a PWS (shared supplies) the measured value was ascribed to all households which were using this water. For repeat or duplicate samples (<1%), we took the highest (worst case) As concentration, although there was little difference in the levels [30].

### 2.5. Transformations of Arsenic Distributions

Water chemistry data, however sampled, do not tend to follow a normal distribution. We intended to find a transformation of the As data collected for which the normal distribution approximation of the transformed data would be most appropriate for each SBGC. Traditionally, the following transformations are considered to “normalize” the data: cubic, square, identity, square root, log, inverse square root, inverse, inverse square and inverse cubic. The “ladder” command in Stata was used to explore the best fit transformation. The fit of the transformed data to normal distribution was then tested by Tukey’s Ladder of Powers [42] and the transformation for which the p-value from the test was the largest was selected as being the best transformation, subject to having at least 30 to 50 collected samples for the SBGC being considered. A graphical indication of the suitability of the normal distribution of each transformation was also employed, namely the quantile–quantile (Q–Q) plot, where deviations of the plotted points from a straight line being indicative that a normal distribution might not be suitable. When a variable is restricted to positive values and can vary over a wide range, it is often the situation that the logarithm of the data can be approximated by a normal distribution [43], in which case the data are said to follow a log-normal distribution. Concentrations of chemicals in environmental media often fit a log normal distribution [37,44] and have been used to model radon concentrations in indoor air [39]. This is also the case for As in, for example, DW [45,46], stream water [47], soil [48,49] and biomonitoring samples [50].

We explored the distribution of samples and calculated the geometric mean and standard deviation, examined the potential for fitting a log normal distribution and explored extremes. For SBGC with a small sample size (n < 30) or where there was no suitable transformation, we assumed log normality as that distribution was identified as the best fit for SBGC with a larger number of data points.

### 2.6. Geological Differences of Arsenic Distribution

To test the need for a model for As concentrations on each geology, we compared the log transformed distributions to assess whether they differed by SBGC. First, we tested the assumption of homoscedasticity (uniformity of variances) by using two variations of Levene’s test [51], both replacing the mean; one with the median and the other with the 10% trimmed mean. If the variances could be considered equal, then a *t*-test [43] for difference between means was applied, otherwise the Kruskal–Wallis rank sum test [43] was used to compare medians.

### 2.7. Hazard Assessment Model

Using the log transformed values, represented by the geometric mean and geometric standard deviation, we calculated the cumulative frequency distribution curve in MS Excel for PWS DW As on each SBGC. The probability (and proportion (%)) of dwellings that occurred in each As exposure category of interest was calculated; i.e., low (<1 μg/L), medium (1–5 μg/L), high (5–10 μg/L), and over the PCV (≥10 μg/L).

We compiled a hazard ranking tool in MS Excel to predict the number of dwellings in each exposure category, given the total number of dwellings with PWS on a SBGC. The exposure categories were as follows:

- More than 10% of the dwellings over the PCV was highest risk-rank 1;
- 5–10% of dwellings over the PCV-rank 2;
- 1–5% of dwellings over the PCV-rank 3;
- Less than 1% of dwellings over the PCV was the lowest risk-rank 4.

Rural land use was found to be a good indicator for the location of PWS, as 97% of PWS in Cornwall were in ‘rural’ areas according to the UK Office of National Statistics (ONS) Urban-Rural (2011) [52] land use classification for Census Output Areas (OAs) [31]. By overlaying the SBGC with the rural land use classifications and residential population layers in the GIS, we could predict the potential number of dwellings with PWS per SBGC. This was fed into the hazard ranking tool.

## 3. Results

### 3.1. Study Population and Arsenic Concentrations Measured

Of the Cornish population of 545,335 in 2014 [53], the Drinking Water Inspectorate (DWI) (2015) [1] estimated that 5.25% were served by 3811 PWS, considering all types of PWS, implying that approximately 28,630 people in Cornwall are drinking water from PWS [31].

From the ~2000 invitation letters sent out, around 25% of households took up the offer of water testing; 497 PWS were sampled in 2011–2013 across the SBGC (Figure 1). From the telephone survey, 516 dwellings were recruited and on interview were found to use the 497 PWS sampled. However, eight households did not use the water for drinking purposes. This left 508 domestic dwellings qualifying for inclusion in the study.

Measured As concentrations in DW ranged from 0.2 to 435 μg/L with a median of 0.37 μg/L, 25th percentile of 0.15 μg/L and 75th percentile of 1.46 μg/L (Table 1) [30]. Nearly 6% of sampled households utilised DW from PWS with As concentrations exceeding the PCV of 10 μg/L. A further 4% of households recorded PWS As concentrations between 5 and 10 μg/L, 30% of households had PWS As concentrations between 1–5 μg/L, and 60% were below 1 μg/L [31]. Descriptive statistics of the measured As concentrations per geological classification are shown in Table 1. The number of samples per SBGC ranged from 3 to 93 and on the mineralised zone, n = 140. The highest As concentrations were found on the Lower Carboniferous SBGC (group 4), with 20% of dwellings that were tested on this geology failing the PCV.

### 3.2. Transformations of Arsenic Distributions

The best fit transformation of the data to normality was identified using Tukey’s ladder of powers based on the largest p value and lowest chi-squared value (Table A1). For SBGC groups 2, 5 and 6 a logarithm was the best transformation. SBGC groups 3, 4, 8, 9 and 10 did not have the minimum number of observations to reliably find the transformation. For groups 1 and 7, normality was not appropriate for any of the transformations. For these and the SBGCs with insufficient observations (n < 30), we assumed a log transformation (Table A1) as this was consistent with other studies of As in environmental samples [45,46,47,48,49,50]. In exploring the fit of log normality to the distribution of As per SBGC, the visual Q–Q plots suggested that many of the groups could be transformed to normality using a log transformation, but variation at the upper bounds of the quartiles sometimes deviated from the lines (Table A1). However, as described in the methods (see Section 2.5) log normality was chosen as best fit, so in examining the distributions, we calculated the median, 25th and 75th percentile values, minimum, maximum, the geometric mean and geometric standard deviation which better described the variation per SBGC (Table 1).

### 3.3. Geological Differences of Arsenic Distribution

To compare distributions between SBGC using parametric statistics, we tested the assumptions upon which these are based. Using Levene’s robust test statistic for the equality of variances between the groups, we found that there was heteroscedasticity (*p* < 0.001). Therefore, we applied the Kruskal–Wallis rank sum test, which demonstrated that the As distributions were significantly different (*p* < 0.001) across SBGC. The medians ranged from 0.08 to 0.5 μg/L on different SBGC.

### 3.4. Hazard Assessment Model

Applying the log-normal distribution identified above as an appropriate mathematical method for describing the distribution of As in PWS in Cornwall, we modelled As levels per SBGC by using the geometric mean and geometric standard deviation. Using the cumulative frequency distribution, the probability (and proportion) of dwellings falling within the exposure categories of interest was obtained (Table 2). This was converted into the percentage of dwellings that were predicted to be in each exposure category. Using the proportion predicted to exceed the PCV, which ranged from 0 to 20%, we ranked the SBGC according to the risk of high As exposure. SBGC 4 was ranked highest (1), SBGC 1, 5 and 9 ranked second, SBGC 6 third and finally SBGC 2, 3, 7, 8 and 10 were lowest risk (rank 4) (Table 2).

The hazard ranking tool rendered in Microsoft Excel allows the user to input the number of PWS on a SBGC and the number of dwellings predicted to fail the PCV on each SBGC is calculated. For example, if 200 dwellings on each SBGC were identified to have PWS, 96 in total were predicted to fail the PCV for As (5%), with the most occurring on lower Carboniferous and volcanic geology (Group 4) (n = 41, 43% of the total dwellings failing the PCV) (Table S1). 

## 4. Discussion

This paper describes a hazard assessment method to rank hazard according to potential for As concentrations in water by simplified geological classifications. Arsenic exposure varied across SBGCs. Dwellings on the Lower Carboniferous and Volcanics geology (Group 4) were associated with the highest potential for elevated As above the PCV. The method developed can be applied to assess and rank the geological hazards in other areas where As concentrations have been measured on different geologies. The design of the sampling was based on characterising exposure for a population-based risk assessment with public health perspectives.

### 4.1. Chemical Hazards in PWS

While the acute health effects of human consumption of As-contaminated water are well documented [54,55], the preventable public health burden from acute As exposure is mainly confined to historical incidents in the UK. Extensive delivery of public water supplies to most of the country and robust arrangements for monitoring and controlling DW standards in supplies [1,28,33,56] limits contemporary exposure to As. There is a wealth of evidence and published research on the carcinogenic properties of As and the effects of sustained uptake through this pathway [6,7,57,58,59,60,61]. 

The hazard assessment showed that As exposure varied across SBGCs with dwellings on the Lower Carboniferous and Volcanics geology (group 4) posing the highest hazard for As concentrations. SBGC alone does not predict GW As hazard; having As rich soil or mine spoil influencing borehole, GW or run off may contribute to elevated As concentrations in water among other factors (e.g., hydrology, well depth, rock structure, etc.) [27,61,62,63,64,65,66,67,68]. For this study, 6% of dwellings with PWS in Cornwall were previously reported by Ander et al. [30] and Crabbe et al. [31] to have elevated As above the PCV. Therefore, posing a risk of health impacts known to derive from As exposure, e.g., developing skin, lung, and/or bladder cancer, peripheral vascular disease, peripheral neurotoxicity and adverse foetal effects [10,69,70], skin disorders, cardiovascular disease, diabetes, and perinatal outcomes [6,7,57,71,72].

Arsenic is not the only chemical hazard potentially present in PWS. In Cornwall, Ander et al. [30] also reported exceedances of PCVs for pH, manganese, nitrate, aluminium, copper, iron, lead, nickel, fluoride, antimony and cadmium. However, in this paper we focused on As, owing to the incidence of exceedance above the PCV and it is the most concerning in terms of possible health impacts [4].

### 4.2. The Tracking Approach

The goal of an EPHT programme is to identify, monitor and reduce hazards and therefore lead to prevention of chronic disease. This is relevant also to hazards such as contaminants in PWS. Targeted sampling of wells providing PWS is an established practice as part of EPHT programmes in the USA, as it is a more efficient approach to characterisation of population hazard in relation to As [15]. For example, the Florida Department of Health (FDOH) conducts targeted private well testing in areas of concern, in partnership with the Florida Department of Environmental Protection (FDEP), as regulated by Florida Statute. Between 2007 and 2012, the FDOH sampled more than 11,000 wells for As and found approximately 1600 wells with As concentrations above 10 μg/L (this is targeted sampling and does not represent a random sample of wells) [73]. This contrasts with the design that would be usually chosen when characterising SBGC over a large area (e.g., by random sampling) [74].

As both EPHT and geology are complex frameworks involving several disciplines and activities, this study alone could not address all elements of either discipline. However, it is a starting point on which to build other aspects of the EPHT concepts. For example, completing the exposure model, health risk assessment, risk communication and risk management aspects.

One crucial aspect of building the overall EPHT approach to chemicals in PWS included the bedrock as one factor, similar to that applied by others [16,27,67], although in this case a simplified geology approach. Hossain et al. [66] showed the potential for educating local drillers to target safe aquifers on the basis of the color characteristics of sediments to predict risk associated with elevated As in Bangladesh. Ayotte et al. [67] developed a multivariable model for predicting GW As concentrations for exposure assessment. The model included geologic As sources, geochemical processes, hydrogeologic processes and land use as explanatory variables. Some individual bedrock geological units were associated with low As concentrations (e.g., gneiss), while the presence of As concentrations ≥5 μg/L in bedrock wells was associated primarily with local scale geological formations (Eliot, Kittery and Madrid Formations, with up to 13 fold greater odds). Arsenic in stream sediments increased the odds of elevated As concentrations in GW by 67% (*p* < 0.001). Geochemical processes were also shown to be associated with elevated As, e.g., Pleistocene marine inundation and wells within 3 km of intrusive igneous plutons. We observed similar effects with the “mineralisation zone” (Group 1) with elevated As in PWS (mean of 6.46 μg/L).

Katner et al. [16] also reported that poor water quality data and a large number of unregistered wells impeded risk determination of water As hazards in Louisiana, US. Even with limited data, they were able to rank parishes with a sample of 10 observations or more using the 90th percentile of GW As levels. Parishes were prioritised for monitoring and outreach based on a classification of either “potential hazard” of elevated arsenic and/or “high domestic well water use”. Three parishes within Louisiana were identified as high priority as they had both identified characteristics. Data were evaluated to identify patterns that might suggest additional areas for targeted outreach and monitoring. Through EPHT and water initiatives outreach, barriers to private well water testing were identified. Although not based on geology, the Katner et al. [16] study describes an alternative approach to prioritising areas for PWS risk or hazard assessment.

Overall, the PWS activities focused on chemicals, and in particular As, have provided an important proof of concept study for the development of EPHT in England, leading to increased awareness and acceptance of the EPHT approach.

### 4.3. Limitations

Based on the consideration that the population risk from As in PWS can be addressed by a dedicated EPHT programme, the study has some limitations. These include the following: 

1. The majority of DW samples were subject to treatment (89%), being physical filtration (62%) or chemical treatment (47%) [30]. As participation in the study was voluntary, requiring participants active inclusion, supplies tested are probably more likely to be treated. The As concentrations used to build the model are in part a reflection of treatment adequacy rather than untreated borehole or GW concentrations. Although this is a consequence of the design that could be seen as a limitation from the point of view of a purely geological assessment, comparison of paired GW and DW samples in a subset (33%) indicated that As concentrations were very similar in most cases [30]. We chose such approach of a model built on exposure to As concentrations actually occurring for the population, as this is appropriate for an application to EPHT.

2. The relationship between geology and As in water will likely differ from Cornwall compared to other regions. However, Cornwall was previously reported with elevated As levels in DW, provided a high proportion of rural private water supplies and highly variable geology to test an EPHT approach to hazard assessment in private drinking water supplies according to geological classification [30,73,75,76,77,78]. Although the model may be valid in the area, it could easily be developed and applied to other geographical areas at a later stage.

3. The widespread mining activity in south west England resulted in mine spoil being left on the surface [62,63,64,65] allowing rainwater to carry As into the ground, thus geology is not the sole predictor of As [8,64]. Among other predictors, the local hydrogeology, a range of human activities, seasonality and rainfall may well play a role [67]. These could be considered later, as even in the absence of consideration of such other predictors, the effect of bedrock geology on hazard ranking is strong [68].

4. Geologies containing mineralisation may act as a confounder in the relationship between geology and As concentrations [8]. However, we accounted for this with the Mineralisation category (group 1), allowing for transportation of As in the environment by including a 1 km buffer. Where mineralization is mapped, alternative distance buffers could be explored, such as within 50 m to 500 m. Linear regression could be used to explore relationships between distance from a mineralised vein and transformed As concentrations in PWS.

5. Other than explicitly considering a Mineralisation category, we did not model spatial variation of As hazard within each bedrock geology. In radon mapping, PHE found that radon potential varied within areas shown on the geological maps as a single uniform geological unit [79] and the association of higher arsenic groundwaters with mineralization in this study similarly reflects the potential considerable lateral and vertical intra-unit variation of groundwater chemistry. In this study, we did not collect sufficient samples to allow us to plot the variation of As within each SBGC. Mapping on the basis of uniform geological groups for population level assessment was a first step in the analysis of these data. Further geostatistical analysis may provide insight into such variations, although ultimately the multi-scale complexities of hydrolithological heterogeneities might limit the cost-effective utility of more geologically detailed-based models.

6. Our model was limited by the number of samples in some SBGC and in those circumstances where a unit had fewer than 30 samples we assumed log normality for the reasons mentioned above, where in reality a different distribution may prevail. However, others who examined As distributions in water and soil have found the log-normal distribution to fit with a relatively small sample size (e.g., n = 33 [37], or n = 30 [47,48,79]). Log normality was a good fit for the majority of data points from Q–Q plots of the data. Therefore, the geometric means and geometric standard deviations better represented the central tendency of the distribution than the arithmetic mean and arithmetic standard deviation, so we used the former to model the distributions. Although the (geometric) mean (GM) and standard deviation were calculated from the collected samples for that geology, for those geologies with few samples, the confidence intervals (CI) of those quantities would be very wide (e.g., for group 8 GM 95% CIs are 0.006 to 1.103 μg/L) which in turn would greatly affect the proportions in each of the exposure categories and therefore the ranking. This means that the results for these geologies are less reliable than those with rather more samples.

7. Exposure to As in householders with PWS also depends upon the amount of water consumed, length of residency, seasonality of residency (e.g., permanent versus summer residents) as well as exposures through other routes (food, soil, air, dust) as explored in Cornwall and elsewhere in south west England [34,35,62,76,77]. Urine, hair and toenail samples taken from 127 households of the same cohort of PWS users indicated that As exposure via DW is likely a dominant source [34,35] as found by others [6,36,59,68,73,78,80,81,82].

The above considerations illustrate the limitations in applying this hazard assessment method as a basis for advice to regulators. We estimate likely dwelling numbers and proportions at risk and how to best find those at risk. However, some of the methods described will introduce bias, probably increasing the prediction of the scale of the risk (worst case estimate). Our results are being considered by Cornwall Council for application to their PWS management programme. Also, this paper reports on a methodology that might be applied in other regions, perhaps adapting hazard models according to the local geology. For example, the model could easily be applied to the neighbouring county of Devon, some 4580 km^2^ that share nine of the same SBGC geologies, covering ~70% of the land area.

### 4.4. Communication

The EPHT approach supports monitoring, surveillance and evaluation of interventions to minimise environmental exposures and effects on health. Ultimately, for small domestic unregulated PWS on higher risk geologies, owners should be encouraged to test for arsenic, and other chemicals with health based PCVs, in PWS and maintain or put in place effective water treatment systems [4]. Targeted interventions should address social vulnerability factors (e.g., income and education) that influence uptake of testing [17,18,19]. Considerable effort was made in communicating the results of the testing back to PWS users for this study and preparing public health and mitigation advice [31,34]. Fact sheets on the chronic health effects of As and other chemical exposures were devised and a programme of stakeholder and householder communications was essential to support the research [83].

Specific treatment can lower concentration of As and residents are advised to test regularly for As and regularly maintain any water filtration and treatment equipment. PHE produced advice for members of the public about As exposure [83,84]. Cornwall Council now require testing for As in PWS, as it is a known risk in the county. In practice, some, but not all, householders took action to lower their exposure to As in their PWS by maintenance of treatment systems or switching to bottled water [34].

### 4.5. Implications of This Work and Next Steps

Strengths of this approach allow for the identification and quantification of the populations affected and provides good evidence for allocating and prioritising precious public resources. For example, if a local authority were to test 500 PWS in a Lower Carboniferous and Volcanics geology area, the best estimate from this work is that they would find approximately 100 dwellings having DW over the PCV. If they tested 500 PWS, using this method of prioritisation by SBGC, say in hazard ranking groups 1 and 2, they would capture nearly half (45%) of all of the exceedances occurring in the area. Overlaying the high As risk SBGCs with another predictor of exposure, such as rural land uses (and/or population density), would prioritise areas where further exploration of As risk should occur.

Using a smaller distance unit for exposure related to distance from mineralisation (group 1) may provide a more accurate reflection of the relationship. It seems likely that a combination of distance methods and SBGC would provide more accurate indications of arsenic potential. Further development of the model would also need to consider the relationship with GW samples and geology, as well as additional consideration of other predictors of arsenic risk, e.g., sediment colour, well depth, treatment methods, rock structure, geochemical and hydrogeologic processes and land use as explanatory variables, alongside validation and testing in other areas. A model using geology and mineralogy (e.g., sulfide minerals) of the bedrock is an important first approach for tracking As in groundwater.

The model needs ground-truthing or independent validation by testing in areas not sampled but with similar geologies, such as in Devon, also in south west England. Taking the same approach, the methodology can be applied and expanded to other areas, by assessing the geological classifications and hazard according to those in each region. There is likely to be some commonality with similar geologies. Applying simulation techniques such as bootstrapping or Monte Carlo methods [39] to various distributions can test the model’s robustness. However, a further sampling campaign will be needed to test the estimated distributions. Alternatively, local authorities and regulatory bodies, such as the Drinking Water Inspectorate (DWI), can be encouraged to collate and provide data or co-ordinate data collection in order to test this approach.

This paper describes just one approach for hazard assessment as part of a risk assessment of the health effects of chemicals in PWS [6,85]. Exposure assessment also considers the time and location of exposure, route of entry, study population, magnitude of expected risk, relation between external exposure and biological response, and other contributing and modifying factors (e.g., social economic status, deprivation, occupation) [86]. Nieuwenhuijsen [87] defines a hierarchy of exposure data and surrogates based on the quality and type of available data, although the role of integration of several data sources for one valid exposure model is important. Risk assessments often make use of information that is cost-effective to collect and available within the confines of a study. Surrogate data for characterising exposures often use proxies for “residence or employment in the geographical area in reasonable proximity to the site where exposure can be assumed”. Here we use residence on a particular geology to assume a level of As exposure through PWS. Distance to a mineralised area may also approximate exposure as shown here for patterns of As within 1 km from a mineralised vein or area.

Local authorities may be able to use this method to contribute to the risk assessments required by legislation [28,33]. In the case of a supply provided to a single dwelling, the duty applies only where local authorities are requested to do so by the owner or occupier of a dwelling. The risk assessment must establish whether there is a risk of supplying water that would constitute a potential danger to human health [28]. With further development and testing of validity, the model can calculate the number of dwellings affected by elevated As, help prioritise areas for risk assessments, sampling and can help target areas for public information campaigns. As it stands, the limited sample sizes in some SBGCs limits the robustness of the model, but local authorities and other agencies will be able to test the model to build the rationale for its application.

Local authorities in the UK assess the hazard associated with PWS as required by national regulations [28,33], which was implemented from the EU Directive 98/83/EC [88]. Installation of chemical treatment can decrease As concentrations to levels below the PCV. However, the authority responsible for reviewing the need for risk assessments is the DWI.

## 5. Conclusions

For this study, the hazard assessment showed that As concentrations varied across SBGCs with dwellings on the Lower Carboniferous and Volcanic geology posing the highest hazard for elevated As above the PCV of 10 μg/L in PWS. Concentrations >10 μg/L increase the risk of developing skin, lung, bladder cancers and several other health impacts known to derive from As exposure.

Within each simplified geological area, concentrations of As in PWS were found or assumed to have log-normal distributions. Across the same areas, the proportion of dwellings predicted to have DW over the prescribed concentration value (PCV) for As ranged from 0% to 20%. From these results, a pilot predictive model was developed calculating the number and percentage of PWS falling within exposure level categories or above the PCV for As. Local authorities may use this to inform PWS risk assessments. The hazard rating will help local authorities to make an informed decision on the need to sample for As.

The geological and GW conditions that promote high arsenic concentrations are known and can help identify high-risk areas for public health [5,8]. Geology varies from surface to depth, therefore, characterising areas by bedrock geology evident in surface mapping also results in oversimplification. However, we found that utilising SBGC to characterise a specific region (Cornwall) for population level exposure groups and exposure to As was helpful to identify significant variation between geologies. 

For future work, this paper highlights the potential for low-cost hazard assessment of As in PWS according to SBGC, which can be adjusted to specific regions. There is also the potential to model distributions of other chemicals in PWS and define a similar hazard assessment tool using SGBC along with other predictors, although this will require some adaptation of the model, alongside validation and testing in other areas. A model using mineralogy (e.g., sulfide minerals) of the bedrock would be an important approach for tracking As in groundwater in other areas.

This work can help to support the development of public health risk management, communication and other interventions for authorities in addressing the chemical quality of PWS. Ultimately, users of PWS should be encouraged to test for As and other chemicals if they are at risk, and maintain water treatment to lower consumption of potentially health threatening contaminants. Furthermore, As concentrations can change in any given well over the course of a few years so that regular monitoring is suggested in high-risk areas [8].

## Figures and Tables

**Table 1:** Descriptive statistics of arsenic concentrations (μg/L) in Private Water Supplies (PWS) in dwellings classified by Simplified Bedrock Geological Category (SBGC) measured in Cornwall, UK.

Geology/Rock Type (SBGC)	Number of Dwellings Sampled	Percentage of Total Dwellings	Arithmetic Mean As	Median As *	Minimum As ^$^	25% Tile	75% Tile	Maximum As	Geometric Mean As	Geometric Standard Deviation As	% of Dwellings Measured at Levels: (As μg/L)			
											<1	1–5	5–10	>10
01 Mineralised	140	28	6.46	0.71	0.02	0.22	2.67	231	0.885	1.805	58	27	6	9
02 Granites and felsic intrusions	93	18	1.24	0.5	0.02	0.26	1.13	20.5	0.575	1.214	71	27	1	1
03 Bude and Crackington Formations	11	2	0.12	0.09	0.02	0.04	0.17	0.37	0.086	0.885	100	0	0	0
04 Lower Carboniferous and Volcanics	19	4	33.13	0.4	0.05	0.25	4.37	435	1.213	2.542	58	21	0	21
05 Middle and Upper Devonian, with Tamar Gp	73	14	5.08	0.42	0.02	0.18	1.97	178	0.622	1.819	68	15	11	5
06 Meadfoot and Dartmouth Formations	69	14	1.51	0.38	0.02	0.12	1.19	14.2	0.441	1.577	68	25	3	4
07 Gramscatho Formation	66	13	0.40	0.17	0.02	0.09	0.32	5.13	0.175	1.109	94	3	3	0
08 Minor Basic and ultrabasic intrusions	3	<1	0.12	0.08	0.03	0.03	0.24	0.24	0.083	1.040	100	0	0	0
09 Mylor Slate Formation	18	4	4.64	0.38	0.06	0.15	3.61	25.6	0.665	2.024	72	6	6	17
10 Lizard and Start Complexes	16	3	0.33	0.13	0.04	0.12	0.15	3.29	0.141	0.977	94	6	0	0
Non-mineralised (groups 2–10)	368	72	3.63	0.31	0.02	0.13	0.92	435	0.411	1.637	76	17	4	4
All	508	100	4.41	0.37	0.02	0.15	1.46	435	0.508	1.718	71	19	4	6

* Kruskal–Wallis test result comparing medians *p* < 0.001. ^$^ Limit of detection is 0.02 μg/L.

**Table 2:** Data transformations; summary statistics of logged transformed values, probability of dwellings being in an exposure category and hazard ranking of Simplified Bedrock Geological Classifications (SBGC).

		Log Transformed Data		% of Dwellings over the Geological Groups Predicted at Levels: (As μg/L)				Hazard Ranking
Geology/Rock Type (SBGC)	Number of Dwellings Sampled	Geometric Mean	Geometric Standard Deviation	<1	1–5	5–10	>10	
01 Mineralised	140	0.885	1.805	52.7	30.4	7.91	8.95	2
02 Granites and felsic intrusions	93	0.575	1.214	67.6	28.7	2.81	0.93	4
03 Bude and Crackington Formations	11	0.086	0.885	99.7	0.28	0	0	4
04 Lower Carboniferous and Volcanics	19	1.213	2.542	47.0	24.2	8.54	20.3	1
05 Middle and Upper Devonian, with Tamar Gp	73	0.622	1.819	60.3	27.1	6.25	6.34	2
06 Meadfoot and Dartmouth Formations	69	0.441	1.577	69.8	24.0	3.79	2.38	3
07 Gramscatho Formation	66	0.175	1.109	94.2	5.67	0.11	0.01	4
08 Minor Basic and ultrabasic intrusions	3	0.083	1.040	99.2	0.73	0.07	0.04	4
09 Mylor Slate Formation	18	0.665	2.024	58.0	26.1	6.92	9.03	2
10 Lizard and Start Complexes	16	0.141	0.977	97.8	2.23	0.01	0	4

## Supplementary Materials

The following are available online at www.mdpi.com/1660-4601/14/12/1490/s1, Table S1: Tool for calculating the number of dwellings exceeding the PCV in each exposure category.

## Appendix A

**Table A1:** Summary of arsenic concentration distributions per Simplified Bedrock Geological Classification (SBGC); exploring distributions, log-normality and transformations.

			Log-Transformed Data		Assumption Made for Distribution
Geology/Rock Type (SBGC)	Number of Properties Sampled	Transformation for Normal Distribution	Q–Q Plot	Chi Square Test *p* Value *	-
02 Granites and felsic intrusions	93	log		0.471	Log normal
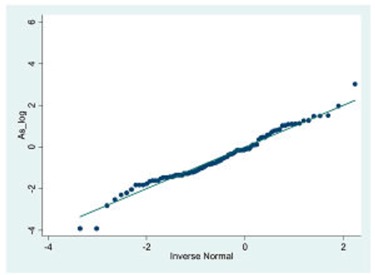
03 Bude and Crackington Formations	11	Insufficient observations		0.861	log normality assumed
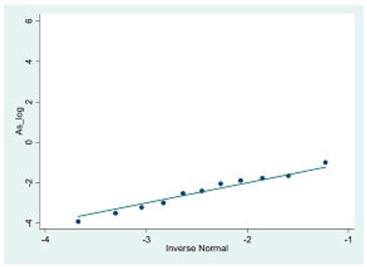
04 Lower Carboniferous and Volcanics	19	Insufficient observations		0.139	log normality assumed
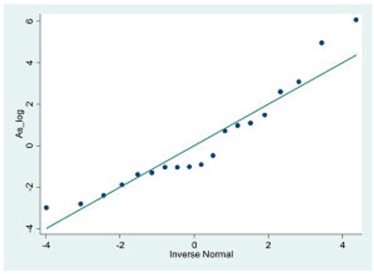
05 Middle amd Upper Devonian, with Tamar Gp	73	log		0.049	Log normal
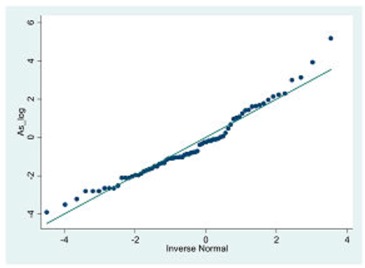
06 Meadfoot and Dartmouth Formations	69	log		0.256	Log normal
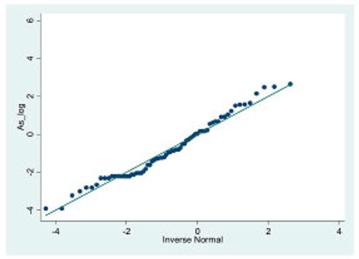
07 Gramscatho Formation	66	none		0.009	Log normality assumed
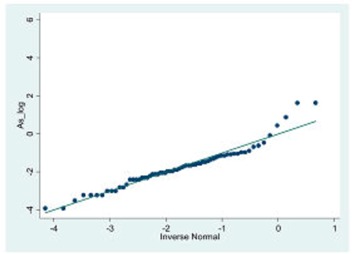
08 Minor Basic and ultrabasic intrusions	3	Insufficient observations		Insufficient observations	log normality assumed
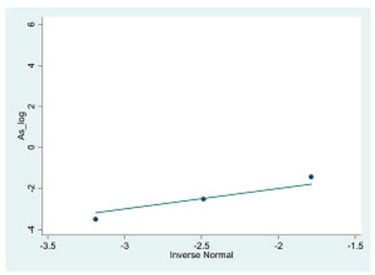
09 Mylor Slate Formation	18	Insufficient observations		0.180	log normality assumed
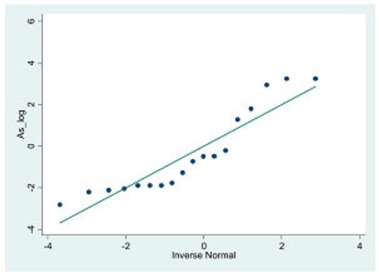
10 Lizard and Start Complexes	16	Insufficient observations		0.0004	log normality assumed
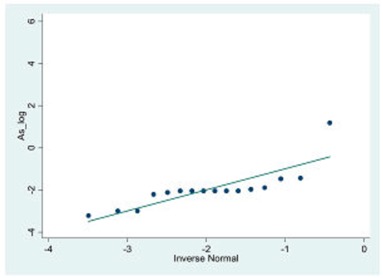
01 Mineralised	140	none		0.017	log normality assumed
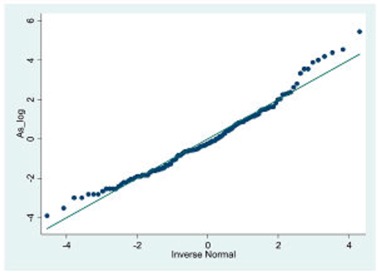
Non-mineralised (groups 2–10)	368	none		<0.001	n/a
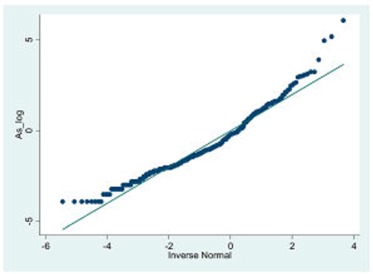
All	508	none		<0.001	n/a
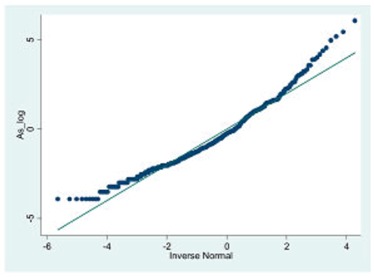

* the null hypothesis is that the data fits the described distribution, so a *p* value of >0.05 suggests this is a good transformation.
