# Supplementary material for: Hazard Ranking Method for Populations Exposed to Arsenic in Private Water Supplies: Relation to Bedrock Geology
Source: Int J Environ Res Public Health. 2017 Dec 1;14(12):1490. doi: 10.3390/ijerph14121490 (PMC5750908; doi:10.3390/ijerph14121490)
Supplement: Supplementary file 1 [file ijerph-14-01490-s001.zip › ijerph-231740-suppl/Table S1- Supplementary Material.docx]

Insert the number of dwellings with PWS on each geology in the yellow cells (200 is shown as an example).

**Table S1:** **Tool for calculating the number of dwellings exceeding the PCV in each exposure category.**

|  |  | **Modelled Distrbution** | | **% of Dwellings Predicted at Levels:  (As µg/L)** | | | | **Input no of Dwellings with PWS on Each Geology *** | **OUTPUT: Number of Dwellings Predicted at Levels** | | | | **Risk Ranking** |
| --- | --- | --- | --- | --- | --- | --- | --- | --- | --- | --- | --- | --- | --- |
| Geology/rock type (SBGC) | Number of dwellings sampled | Geometric mean | Geometric standard deviation | <1 | 1–5 | 5–10 | >10 |  | <1 | 1–5 | 5–10 | >10 |  |
| 01 Mineralised | 140 | 0.753 | 1.805 | 52.7 | 30.4 | 7.91 | 8.95 | 200 | 105 | 61 | 16 | 18 | 2 |
| 02 Granites and felsic instrusions | 93 | 0.280 | 1.214 | 67.6 | 28.7 | 2.81 | 0.93 | 200 | 135 | 57 | 6 | 2 | 4 |
| 03 Bude and Crackington Formations | 11 | 0.004 | 0.885 | 99.7 | 0.28 | 0 | 0 | 200 | 199 | 1 | 0 | 0 | 4 |
| 04 Lower Carboniferous and Volcanics | 19 | 1.560 | 2.542 | 47 | 24.2 | 8.54 | 20.3 | 200 | 94 | 48 | 17 | 41 | 1 |
| 05 Middle and Upper Devonian, with Tamar Gp | 73 | 0.335 | 1.819 | 60.3 | 27.1 | 6.25 | 6.34 | 200 | 121 | 54 | 13 | 13 | 2 |
| 06 Meadfoot and Dartmouth Formations | 69 | 0.151 | 1.577 | 69.8 | 24 | 3.79 | 2.38 | 200 | 140 | 48 | 8 | 5 | 3 |
| 07 Gramscatho Formation | 66 | 0.018 | 1.109 | 94.2 | 5.67 | 0.11 | 0.01 | 200 | 188 | 11 | 0 | 0 | 4 |
| 08 Minor Basic and ultrabasic intrusions | 3 | 0.003 | 1.04 | 99.2 | 0.73 | 0.07 | 0.04 | 200 | 198 | 1 | 0 | 0 | 4 |
| 09 Mylor Slate Formation | 18 | 0.392 | 2.024 | 58 | 26.1 | 6.92 | 9.03 | 200 | 116 | 52 | 14 | 18 | 2 |
| 10 Lizard and Start Complexes | 16 | 0.011 | 0.977 | 97.8 | 2.23 | 0.01 | 0 | 200 | 196 | 4 | 0 | 0 | 4 |
|  |  |  |  |  |  |  |  | Total | 1493 | 339 | 73 | 96 |  |

* input the number of dwellings with PWS in these cells per SBGC.
